# Distributed Wireless Monitoring System for Ullage and Temperature in Wine Barrels

**DOI:** 10.3390/s150819495

**Published:** 2015-08-10

**Authors:** Wenqi Zhang, George K. Skouroumounis, Tanya M. Monro, Dennis K. Taylor

**Affiliations:** 1 Institute for Photonics and Advanced Sensing and School of Physical Sciences, the University of Adelaide, Adelaide 5005, Australia; E-Mail: wenqi.zhang@adelaide.edu.au; 2 School of Agriculture, Food and Wine, the University of Adelaide, Waite Campus, PMB 1, Glen Osmond 5064, Australia; E-Mail: george.skouroumounis@adelaide.edu.au; 3 The University of South Australia, Adelaide 5000, Australia; E-Mail: tanya.monro@unisa.edu.au

**Keywords:** wine, wireless, sensor network, temperature, ullage, wine sensing

## Abstract

This paper presents a multipurpose and low cost sensor for the simultaneous monitoring of temperature and ullage of wine in barrels in two of the most important stages of winemaking, that being fermentation and maturation. The distributed sensor subsystem is imbedded within the bung of the barrel and runs on battery for a period of at least 12 months and costs around $27 AUD for all parts. In addition, software was designed which allows for the remote transmission and easy visual interpretation of the data for the winemaker. Early warning signals can be sent when the temperature or ullage deviates from a winemakers expectations so remedial action can be taken, such as when topping is required or the movement of the barrels to a cooler cellar location. Such knowledge of a wine’s properties or storage conditions allows for a more precise control of the final wine quality.

## 1. Introduction

Over the past decade, the development and integration of wireless sensor networks (WSN) within the agriculture and food industry, along with a greater understanding of the theory and potential application of such devices, has seen considerable growth [1,2,3,4,5,6,7,8,9]. Indeed, there is now a multitude of devices designed to provide information on precision agriculture, environmental monitoring, machine and process control, facility automation, food packaging, food inspection and quality control [3,7].

Surprisingly, the use of WSN in vineyards and wineries is still quite rare. In terms of vineyard monitoring, several reports have appeared recently detailing the use of WSN to monitor not only a vineyard’s microclimate but also the risk of vine damage due to frost, pests and disease [10,11,12]. There have also been several reports utilising WSN to monitor the quality of wine in terms of cellaring [13], and temperature control at the various stages of vinification [14,15]. Controlling the temperature during primary fermentations of wine is extremely important in terms of the development of the “bouquet” or “aroma” of a wine. For example, white wines are usually fermented at around 15 °C with higher temperatures (e.g., 20 °C) potentially resulting in the loss of volatile “aroma” due to the sweeping away of these volatiles by the carbon dioxide gas generated during fermentation [16]. In addition, the control of the temperature of wines undergoing malolactic fermentation or maturation in barrels is vital to ensure the control of potentially unwanted bacterial growth or oxidative damage [17]. Currently, the temperature of wines in barrels is simply measured with a thermometer, which means the barrels need to be opened and potentially exposed to oxygen, whilst the temperature is controlled by simply moving the barrels from one cold room to another.

Another key parameter that is monitored for wines in barrels is the extent of ullage. Ullage is defined as the empty space that lies between the wine and the closure, *i.e.*, the space between a bung in a barrel and the surface of the wine, or the space between a cork or screw cap in a bottle and the surface of the wine. Given that this air contains around 20.95% oxygen, minimising ullage is very important to avoid chemical oxidative damage or bacterial damage of the wine [18]. For example, acetobacter (acetic acid bacteria) which converts ethanol to acetic acid is facilitated by the presence of oxygen thereby increasing the volatile acidity of the wine [19]. To our knowledge ,there is yet to be any report on the development of a WSN to monitor ullage.

In this paper, we demonstrate a WSN platform that can be embedded inside wine barrels bung and used to monitor the temperature and ullage of the wine in each individual barrel during both the fermentation and maturation stages of winemaking. Similar pioneer works of potential for the deployment of WSN in wineries have been reported in recent years [20,21]. However, the size, cost and energy consumption are still not ideal for large scale winery use. Our design is particularly focused on these aspects. For instance, Di Gennaro reported that the basic components of the WineDuino node (excluding the actual sensors) cost more than 90 euros each [21]. The aim was to design a device that was of low cost, low-energy consumption and could be used in large barrel rooms containing thousands of barrels with the information being sent to remote computer workstations or a winemaker’s iPhone for consideration. This early warning system thus allows a winemaker to make immediate modifications to winemaking processes to ensure the highest quality wines are being produced. The “smart-bung” and WSN platform developed also allows for the addition of other sensor modules in the future in order to extend its functionality and allow for even closer monitoring of all important analytes and parameters of wine during the vinification and maturation stages of winemaking.

## 2. The Overall Systems Architecture

**Figure 1:**
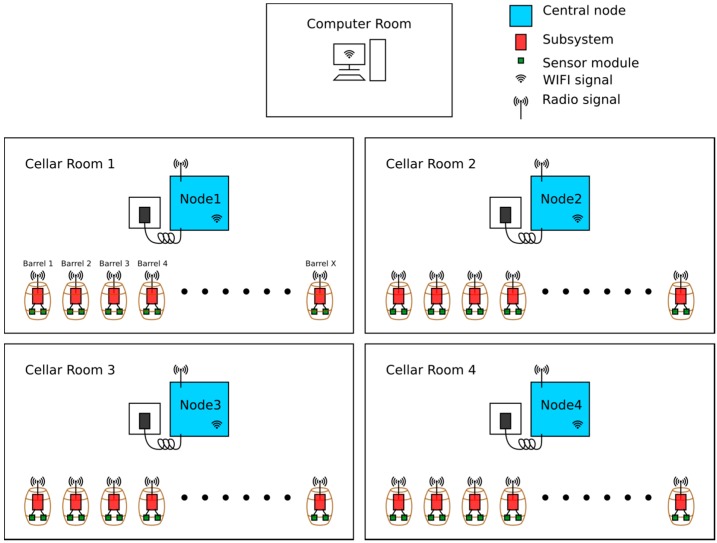
The schematics of the distributed wine monitor system.

The distributed wine monitoring platform consisted of a small central node and a subsystem where sensor modules may be attached, a schematic of which is depicted in Figure 1. The subsystem was an energy-saving small signal chip computer with an on-board FM radio system and general purpose IO interface that could be used to connect to the sensor modules. The subsystem and the sensor modules were to be powered by a long-life battery and consume no energy when in an idle state (most of the time), whilst the subsystem and the sensor modules attached would only be powered on when in data requisition mode. This ensures that the subsystem would run on a single battery for an extended period of time (12 months) without recharging of the battery. The subsystem was small enough that it could be embedded into a barrel bung. The central node was to be built upon a single computer board running a custom build standard Linux operation system which can support running a wide range of programs for gathering and processing the data received from the subsystems. The central node also had the capability of receiving and transmitting both FM radio and WIFI signals. It should be noted that the FM radio modules would consume much less power when compared to WIFI technology which would be used in the central nodes to communicate with the remote computer center.

The subsystems acquire data, push it to the central node and power off immediately at a fixed interval. The central nodes receive the data from the subsystems, identify the source barrels of the data, conduct preliminary data processing and upload the data to the server in the computer room. The server logs and analyses the data, puts them on the web interface and sends out alerts when necessary.

**Figure 2:**
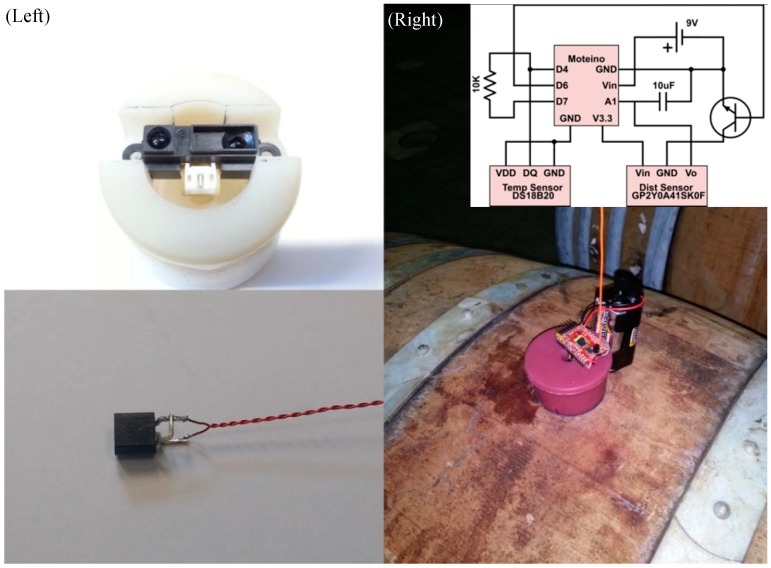
(**Left**) The distance sensor inside a bung and a naked temperature sensor; (**Right**) An assembled subsystem on a wine barrel and its schematic as the inset.

Specifically, a FriendlyARM Mini210s single computer board computer was used as the basis of the central node whilst an ATMega328 chip based Arduino-like single chip microcontroller (Moteino) was used as the basis of the subsystem. An additional Moteino chip was connected to the Mini210s via a USB port serving as a FM radio transceiver. Currently, the Moteino software supports 65,280 chips in its networks, but there is no hardware limitation. The ullage and temperature modules were built using a SHARP GP2Y0A41SK0F (measures 4–30 cm) infrared distance sensor and Dallas DS18B20 1-wire temperature sensor (measures from −55 °C to +125 °C with ±0.5 °C from −10 °C to +85 °C), respectively. Figure 2 depicts the distance sensor assembled in a barrel bung (top left) and an unprotected temperature sensor (bottom left). The temperature sensor was protected with a heat conducing shell and hanging from the bung into the wine. The entire assembled subsystem is depicted in the right photo of Figure 2 and had a total cost of $27 AUD retail price, which could be further scaled down if the system was produced at scale.

In the work by Sainz *et al.*, bluetooth was used as the wireless network interface and an analogue temperature sensor TM35DZ for temperature measurement. Such modules suffer from several drawbacks including that the usual transmission range for bluetooth devices is only a few meters which makes the monitoring system unsuitable for practical use in large wineries where the barrels are constantly being moved around, whilst the accuracy of the analogue temperature sensor would not be as good if a digital temperature was used [14]. Consequently, it appeared to us that our system, which employs a Moteino microcontrollor with an integrated low power FM radio as the sensor platform and main network interface along with a digital temperature probe, will offer additional advantages over what has thus far been reported. Such a radio system would have a coverage of 300–400 m radius at the BAUD rate of 55 kbps (or 1.5 mile range at 1200 bps), more than enough to cover most winery cellar areas. In addition, whilst Boquete *et al.* employed high-precision digital temperature sensors (DS1631 ±0.5 °C from 0 to 70 °C) and an XBee wireless interface with a range of 20–30 m [15], it is now recognised that the price of a XBee wireless module alone is more than the price of an entire microcontrollor plus wireless module, thus our WNS would come in at a much lower cost.

The assembly of the subsystem was rather straightforward. As shown in the top left photo in Figure 2, a slot is cut in the bottom of a bung where the SHARP infrared distance sensor is to be installed. The edge of the distance sensor is coplanar to the bottom of the bung to allow easy measurement of the position of the distance sensor. A small hole is drilled through the bung from bottom to top for any wires including the temperature sensor. The Moteino chip is installed on the top-side of the bung and connected to a battery. The distance sensor and the temperature sensor were soldered onto the Moteino chip according to the schematic shown in Figure 2. A 10 K pull-up resistor was used together with temperature sensor. The 10 μF capacitor between pin A1 and GND was used for reducing voltage fluctuation from the distance sensor. The NPN transistor was used as a switch allowing the program to switch the power to the distance sensor to save energy. The Moteino chip allows the user to use any power supply from 3.3 V to 12 V. We used both packs of 4 AA batteries (6 V) and 9 V batteries to power the subsystem in this work. The central node can be installed anywhere convenient inside the wine cellar next to a power source. Each central node hosts a unique radio network. Each subsystem was pre-programmed with a unique ID and can be assigned to any radio network, which allows each wine barrel to be easily identified by the winemaker. Our design approach greatly reduces the cost of the entire WSN. Table 1 shows the cost of each of the components used.

**Table 1:** The cost of the elements used in this work.

Central Node	
FriendlyARM Mini210S with WIFI	$225.89
Moteino R4 with RFM69WH	$19.95
Total: $245.84 AUD	
**Subsystem**	
Moteino R4 with RFM69WH	$19.95
SHARP GP2Y0A41SK0F	$6.20
Dallas DS18B20	$0.60
Heat Conducing Shell	$0.40
10K Resistor	$0.01
10 uF Capacitor	$0.01
2N2222 NPN Transistor	$0.03
Total: $27.20 AUD	

**Figure 3:**
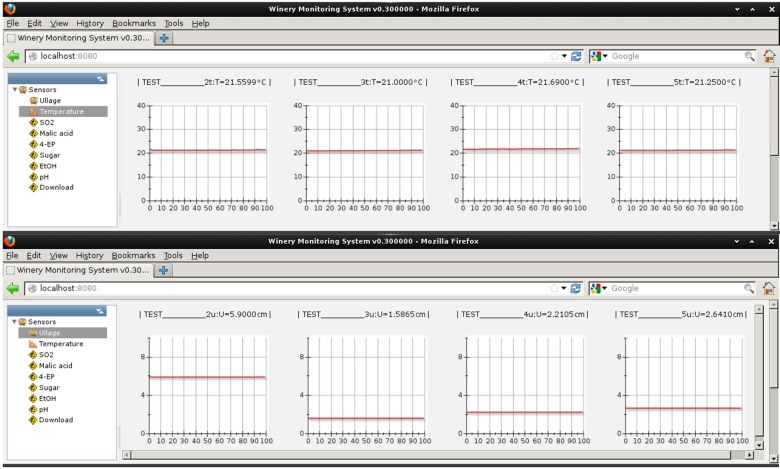
The temperature sensor page of the web interface shows eight virtual barrels.

Specific software was designed which would run on both the central node and computer server. It was written in C/C++ utilizing Qt [22] and Wt [23] packages to ensure portability. The software on the central node collects and processes the sensor data whilst the software on the central computer node serves as a webpage server as shown in Figure 3. The web page consists of two important sections. On the left, the operator can select one of many parameters to be monitored by the “smart bungs”. For example, temperature, ullage, pH *etc*. To the right of this menu is a pane, which displays each individual barrel of wine and graphically or numerically displays what the current status of that parameter is. This setup allows users to access the sensor data using any remote web browser with early warning messages being sent out via emails to the winemakers if pre-set parameters are not being adhered to. For example, if the winemaker wished the wine to be stored in barrels at 15 °C for six months and the temperature deviated by more than 2 °C (pre-set in the software by the winemaker), then an alert would be sent wirelessly to warn of such an instance so remedial action could be taken. The ullage readings were not calibrated to physical units, however, it could be given that the ullage values are inversely proportional to the voltages read from the IR distance sensors. To calibrate the inversed voltage to physical units, one may use the relationship: *L* = *a* * 1/*V* + *b*, where *L* is the distance, and *V* is the voltage, to fit the voltages manually measured for different wine levels to obtain the calibration coefficients *a* and *b*, and input them into the software. The code on Moteino was developed using the Arduino toolkit. Moteino is fully compatible with the Arduino Uno platform.

## 3. Results and Discussion

After building the bungs with associated temperature and ullage sensors incorporated and designing the software, we then tested the system under several real world winemaking conditions. The first was to monitor the temperature and ullage of still wine on the cellar floor over time (maturation in barrels) whilst the second test was to monitor the same parameters during fermentation. In both test trials, four barrels of wine were monitored simultaneously. The sampling rate in our tests was set to once every 8 s for both temperature and ullage.

### 3.1. Trial I—Temperature and Ullage of Still Wine on the Cellar Floor

**Figure 4:**
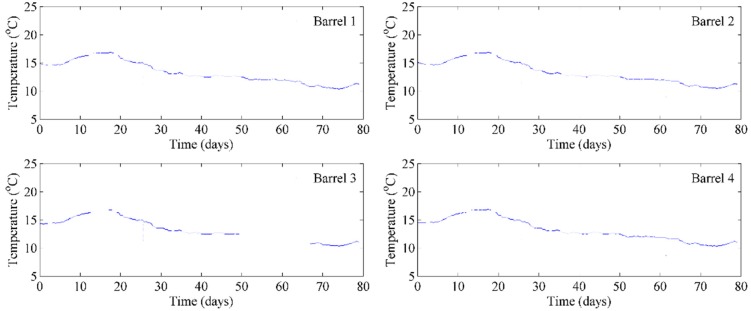
Continual monitoring of temperature of four barrels of wine in storage.

The first trial was performed on four barrels of red wine stored on the cellar floor for a period or nearly three months, where the temperature was not actively controlled, Figure 4. The temperature sensors were placed 30 cm below the bung holes using insulated magnet wires. The four test barrels were set next to each other and were subject to daily variances in temperature in the cellar. It should be noted that day zero was at the beginning of the Australian autumn when the daily temperatures are higher than at the end of autumn when the trial was completed.

It can be clearly seen that the readings from the individual temperature sensors within each bung are remarkably identical except for some minor variations due to small differences in the heat capacity of each barrel. Given that the winemaker wished the wines to be kept in the range of 10–15 °C during maturation, which was observed by our WSN, it provided confidence to the winemaker that the cellar hands did not need to manually measure the temperature of each barrel. On occasions, we opened one of the barrels and manually measured the temperature with a thermometer and found the same values for the temperature of the wine. Furthermore, the barrels did not need to be opened which aids in avoiding potential contamination, and oxidative spoilage. During this first trial, one of the subsystems (bottom left in Figure 4) went offline for about two weeks due to a power glitch. It was recovered by manually rebooting it and could be avoided in the future by installing a “watchdog” timer program, which will automatically reboot the system whenever there is a problem detected.

**Figure 5:**
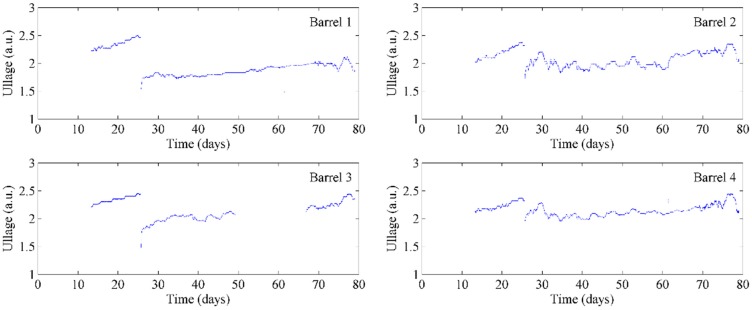
Continual monitoring of ullage of four barrels of wine in storage.

**Figure 6:**
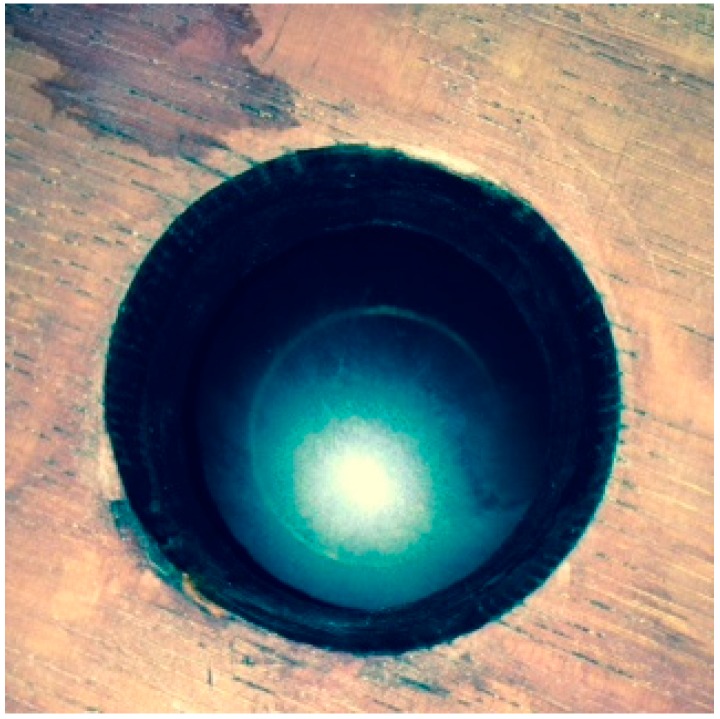
A thin film formed on top of the wine surface that influenced the ullage sensor reading.

At the same time, we also measured the ullage in the four barrels on the cellar floor. The sensors were turned on at day 12 as we found that after fermentation and transfer of the wine into barrels, there was a large amount of foaming that required a number of days to dissipate. The settling down of the wines surface can still be seen during the day 12 and day 25 time-points in the plots of Figure 5. Importantly, all barrels showed a decrease in ullage height at around day 26 which corresponded to the barrels being opened and topped up with additional wine. After this time point, the ullage again began to increase slowly due to slow evaporational losses, as expected. Two of the barrels showed some slight fluctuations in daily ullage levels after topping (right column of Figure 5), which we believe is due to the disturbance of floating films resulting in fluctuations in the ullage depth readings. We inspected one of these barrels and found that there was indeed a thin film floating on the surface of the wine, Figure 6. Thus, given that the sensor we chose for ullage is based on light reflection, consistency of the reflective surface would be extremely important. Any disturbance by floating films would be expected to cause some variation seen in the ullage measurements as found here, although the overall trends found in our trials are what a winemaker would expect to observe. If the wines were to be stored in barrels for extended periods of time (up to two years), as many are, then the winemaker could simply set a minimum ullage distance required before the barrels needed to be topped; when reached, the sensors would trigger an alert that this action needs to be taken.

### 3.2. Trial II—Temperature and Ullage during Fermentation

**Figure 7:**
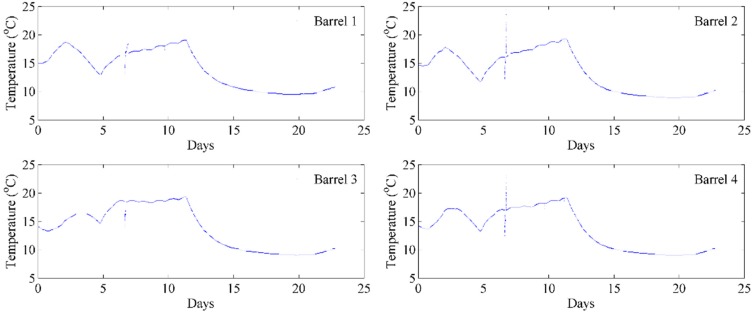
Continual monitoring of temperature of four barrels of wine during fermentation.

Given that our wirelessly distributed system successfully gathered the temperature and ullage information from wines undergoing maturation in barrels on the cellar floor and sent the information to a remote computer server room which can be accessed by winemakers from a simple webpage, we next examined its performance at monitoring temperature and ullage during a real wine fermentation. Again, fours barrels were monitored with the data from the temperature sensors collated in Figure 7. The winemaker instructed the cellar hands to place the juice in barrels in a 15 °C cold room and perform inoculation. We affixed our remote sensors and began monitoring the barrels. Pleasingly, our sensors detected that the juice was around the initial temperature of the cold room, *i.e.*, 15 °C. Naturally, each ferment in each individual barrel will progress through fermentation at slightly different speeds and consequently subtle temperature differences are expected to be observed, as seen in Figure 7. As the ferments began over the first few days, the observed temperature was found to rise several degrees to between 17 °C and 18 °C. Given that the winemaker wished the ferments to be conducted near 15 °C and wished to avoid excessive fermentations of 20+ °C, they made the decision to move the barrels to the 10 °C cold room on the third night. As can be seen, our remote detectors picked this up and the temperatures within the barrels began to decrease by several degrees by day 5. The ferments were then at their full exponential growth phase and thus the temperature began to rise again up to 18 °C as they pushed through to completion over the following five days or so. All the ferments finished around day 12 as the winemaker expected and the temperatures began to drop back to the outside ambient cellar room temperature of 10 °C. They were then moved out of the cold room onto the normal cellar floor for further processing on day 18 which again resulted in a gradual warming of the wine.

**Figure 8:**
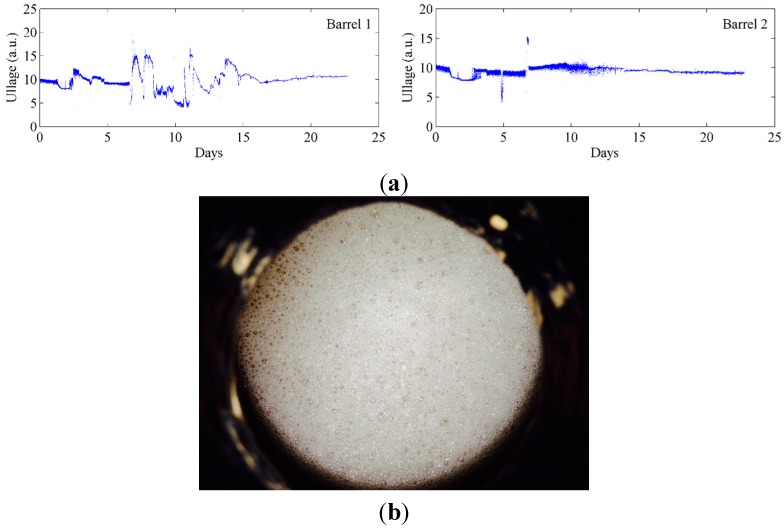
(**a**) Continual monitoring of ullage of two barrels of wine during fermentation; (**b**) the overflowing wine resulting from the excessive bubbling due to carbon dioxide evolution.

At the same time, we also measured the ullage in the barrels during fermentation, the results of which are displayed for two barrels, Figure 8a. Fermentation results in excessive bubbling due to carbon dioxide evolution and in some cases results in the wine overflowing the barrels (see picture in Figure 8b). The bungs come with a hole imbedded in them to allow for such overflows. Given that our ullage sensor is based on infrared light reflection between the surface of the liquid and the bung itself, extensive fluctuation in ullage height was expected as the wines level rises and falls over time. Furthermore, this fluctuation would be exacerbated with changes in the fermentation temperature. Indeed, extensive fluctuations were observed when monitoring the ullage height during fermentation, Figure 8a. After days 12–15, when the fermentations were beginning to be completed and the foams dissipate, the noise in the ullage curves significantly reduces. Importantly, the time-point of 12–15 days indicating completion of fermentation by ullage also corresponds to that observed by our temperature sensors highlighted above. Whilst it was intriguing to measure ullage under real fermentation conditions, the measurement of ullage is of most importance during long-term storage and maturation of wine in barrels.

## 4. Conclusions

In this work, we have designed a simple low-cost energy saving wireless distributed sensor network that allows for the simultaneous monitoring of a wine’s temperature and ullage both in the fermentation and maturation stages of wine making in barrels. The distributed sensor subsystem runs on battery for a period of at least 12 months and costs around $27 AUD for all the parts. In addition, software was designed which allows for the remote transmission and easy visual interpretation of the data for the winemaker, displaying “virtual barrels” that can be monitored remotely. Early warning signals can be sent when the temperature or ullage deviates from a winemaker’s expectations so remedial action can be taken, such as topping up or the movement of the barrels to a cooler cellar. Such knowledge of a wine’s properties or storage conditions will allow for a more precise control of a final wine’s quality. Moreover, the WSN has been designed so that additional modules of analysis (e.g., pH, sulfur levels, ethanol content *etc.*) can be simply added in the future to the “smart bungs” upon development. Finally, we anticipate that in the future these “smart bungs” could be manufactured via the use of 3D printing which may further reduce the cost and allow installation in thousands of barrels in each winery.
